# Epigenetic Regulation and Immune Associations of TRAIL Decoy Receptors TNFRSF10C and TNFRSF10D in Glioblastoma: A Multi-omic Analysis

**DOI:** 10.7759/cureus.105832

**Published:** 2026-03-25

**Authors:** Kartik M Khurana, Ajeet Saoji, Heena Pahuja

**Affiliations:** 1 General Practice, N.K.P. Salve Institute of Medical Sciences and Research Centre, Nagpur, IND; 2 Community Medicine, N.K.P. Salve Institute of Medical Sciences and Research Centre, Nagpur, IND; 3 Anesthesiology, N.K.P. Salve Institute of Medical Sciences and Research Centre, Nagpur, IND

**Keywords:** dna methylation, glioblastoma, immune infiltration, tnfrsf10c, tnfrsf10d, trail signaling, tumor microenvironment

## Abstract

Background

Glioblastoma (GBM) is characterized by immune dysregulation and epigenetic alterations that contribute to tumor progression. Although tumor necrosis factor-related apoptosis-inducing ligand (TRAIL) signaling has been implicated in tumor biology, the transcriptional and immunological relevance of its decoy receptors, TNF receptor superfamily member 10C and 10D (TNFRSF10C and TNFRSF10D), in gliomas remains incompletely characterized.

Methods

An integrative multi-omic analysis was performed using The Cancer Genome Atlas (TCGA), Genotype-Tissue Expression (GTEx), and Chinese Glioma Genome Atlas (CGGA) datasets in combination with Gene Expression Profiling Interactive Analysis 3 (GEPIA3), University of Alabama at Birmingham Cancer database (UALCAN), Tumor Immune Estimation Resource 3.0 (TIMER3.0), Search Tool for the Retrieval of Interacting Genes/Proteins (STRING), and MEXPRESS platforms. Gene expression, promoter methylation, molecular subtype distribution, immune infiltration patterns, and clinical associations were evaluated using harmonized analytical workflows and cross-validation across datasets.

Results

Both receptors demonstrated progressive upregulation from normal brain to lower-grade glioma (LGG) and GBM, with higher expression observed in isocitrate dehydrogenase (IDH)-wildtype tumors. Promoter methylation analysis revealed inverse correlations between CpG methylation and gene expression, suggesting potential epigenetic associations. Immune deconvolution analyses showed consistent associations with myeloid cell populations, including macrophages, neutrophils, and dendritic cells (DCs), alongside limited correlations with T-cell subsets. Protein-protein interaction network analysis indicated that these receptors interact with multiple components of inflammatory and TNF/TRAIL signaling systems. Higher expression showed trends toward shorter progression-free intervals (PFI).

Conclusions

TNFRSF10C and TNFRSF10D demonstrate reproducible associations with methylation patterns and immune microenvironment characteristics in GBM. These findings highlight potential links between TRAIL decoy receptors and inflammatory tumor states and support further mechanistic investigation.

## Introduction

Glioblastoma (GBM) remains one of the most treatment-refractory malignancies in modern oncology [1,2]. Despite extensive investigation into its genomic alterations and therapeutic vulnerabilities, durable disease control remains limited [3]. Increasingly, GBM is recognized not solely as a genetically driven disease but as one influenced by dysregulated signaling pathways, immune dysfunction, and epigenetic alterations [4-6]. These features contribute to its infiltrative growth, inflammatory microenvironment, and resistance to apoptotic and immune-mediated mechanisms [5].

Within this biological context, the tumor necrosis factor-related apoptosis-inducing ligand (TRAIL) signaling pathway presents a notable paradox [7]. TRAIL and its receptors were initially investigated for their ability to selectively induce apoptosis in malignant cells; however, clinical outcomes were limited due to resistance observed in several tumors, including GBM [8]. Among TRAIL pathway components, the decoy receptors TNF receptor superfamily member 10C (TNFRSF10C; DcR1) and TNF receptor superfamily member 10D (TNFRSF10D; DcR2) have received comparatively limited attention. Although classified as anti-apoptotic receptors, their broader relevance in glioma biology has not been comprehensively examined [9]. Existing studies have largely characterized them as inhibitors of TRAIL-mediated apoptosis, rather than evaluating their potential role in the tumor microenvironment [9,10].

The availability of large-scale multi-omic datasets provides an opportunity to reassess this assumption. Decoy receptor expression may be influenced by promoter methylation, inflammatory signaling, and immune cell composition [11]. These factors are particularly relevant in GBM, which is characterized by a myeloid-predominant and relatively T-cell-limited immune microenvironment. However, the behavior of TNFRSF10C and TNFRSF10D across glioma grades, isocitrate dehydrogenase (IDH)-defined molecular subtypes, epigenetic landscapes, and immune infiltration patterns has not been systematically characterized.

In this study, we performed a multi-platform, multi-omic analysis of TNFRSF10C and TNFRSF10D across glioma datasets, with particular emphasis on GBM and IDH-wildtype tumors. By integrating transcriptomic data from The Cancer Genome Atlas (TCGA), Chinese Glioma Genome Atlas (CGGA), and Genotype-Tissue Expression (GTEx) datasets with promoter methylation analysis, genomic alterations, protein-protein interaction networks, and immune deconvolution approaches, we aimed to evaluate whether these receptors are associated with epigenetic regulation and immune microenvironment features in GBM. Our findings provide an integrated overview of the expression patterns and immune associations of TRAIL decoy receptors in GBM and support further investigation into their potential biological relevance.

## Materials and methods

Study design

The primary objective of this study was to evaluate the expression patterns, promoter methylation profiles, and immune microenvironment associations of the TRAIL decoy receptors TNFRSF10C and TNFRSF10D in glioma, with particular emphasis on GBM, using integrated multi-omic datasets.

This study utilized publicly available multi-omic datasets and analytical platforms to evaluate the transcriptional, epigenetic, and immune-related characteristics of TNFRSF10C and TNFRSF10D in glioma, with emphasis on GBM. Analyses were conducted across independent cohorts using complementary platforms. The datasets used for analysis included the TCGA GBM cohort (TCGA-GBM; n = 163), the TCGA lower-grade glioma cohort (TCGA-LGG; n = 518), and the GTEx normal brain tissues (GTEx; n = 207) accessed through Gene Expression Profiling Interactive Analysis 3 (GEPIA3). Independent validation cohorts were obtained from the CGGA, including the mRNAseq_325 dataset (n = 325) and the mRNAseq_693 dataset (n = 693). All datasets were accessed between October and November 2025.

Data sources

TCGA and GTEx

Normalized RNA sequencing data for GBM, LGG, and normal brain tissues were obtained using GEPIA3, which integrates datasets from the TCGA and the GTEx project under a unified processing pipeline (log2(TPM+1)) [12]. Because TCGA contains a limited number of normal brain tissue samples, normal controls were primarily derived from the GTEx project. GEPIA3 integrates TCGA tumor samples and GTEx normal tissues using a standardized processing framework based on the UCSC Xena platform, enabling cross-cohort comparisons of gene expression between tumor and normal tissues.

CGGA

Gene expression data and clinical metadata, including isocitrate dehydrogenase (IDH) mutation status and World Health Organization (WHO) grade, were retrieved from the CGGA mRNAseq_325 and mRNAseq_693 datasets [13]. These datasets were used for IDH-stratified expression analysis and survival validation.

Differential expression analysis

Tumor versus normal comparisons were performed in GEPIA3 using default analysis of variance (ANOVA) parameters (|log₂FC| ≥ 1, q < 0.05). Expression trends across normal brain tissue, LGG, and GBM were evaluated using the GEPIA3 stage plotting module. Correlation between TNFRSF10C and TNFRSF10D expression levels was assessed using Spearman correlation.

IDH-stratified analysis

IDH-dependent expression differences were evaluated using the CGGA mRNAseq_325 dataset. For each WHO grade (II-IV), gene expression levels were compared between IDH-mutant and IDH-wildtype tumors using the Wilcoxon rank-sum test as implemented within the CGGA platform. This dataset was selected because it provides detailed IDH stratification.

Promoter methylation analysis

Promoter methylation differences between normal brain tissue and GBM were assessed using the University of Alabama at Birmingham Cancer database (UALCAN), which reports Illumina Human Methylation 450 (HM450) beta values [14]. Expression-methylation correlations were evaluated using MEXPRESS, which maps CpG probes across gene loci and calculates Pearson correlation coefficients with transcript levels [15,16]. Only promoter-associated probes (TSS1500, TSS200, and 5′UTR) were considered for interpretation.

Copy-number and mutation analysis

Mutation frequency and copy-number variation (CNV) trends were reviewed using publicly available TCGA visualization tools. These analyses were exploratory and were not included in primary statistical modeling.

Protein-protein interaction analysis

Protein interaction networks were generated using the Search Tool for the Retrieval of Interacting Genes/Proteins (STRING) (version 12) with medium-confidence settings. Evidence sources included curated databases, experimental data, and text mining. First-shell interactors were retained for network visualization [17].

Immune infiltration analysis

Immune cell abundance estimates were obtained from TIMER3.0, which incorporates six deconvolution algorithms (TIMER, CIBERSORT, quanTIseq, EPIC, xCell, and MCP-counter) [18]. Correlations between gene expression and immune cell estimates were extracted from platform-generated output tables. Associations observed across multiple algorithms were reported.

Survival analysis

Progression-free interval (PFI) and overall survival (OS) were analyzed using the CGGA survival module, which applies Kaplan-Meier estimation with log-rank testing. Additional survival visualizations were obtained from TCGA survival tools. Expression groups were dichotomized using median expression unless otherwise specified by the platform.

Statistical analysis

Statistical analyses were performed using the built-in analytical functions of the respective platforms. Differential expression analyses in GEPIA3 were conducted using ANOVA with a significance threshold of |log₂FC| ≥ 1 and a false discovery rate-adjusted q-value < 0.05. IDH-stratified comparisons in CGGA datasets were evaluated using the Wilcoxon rank-sum test.

Correlation analyses between gene expression, promoter methylation, and immune cell infiltration were calculated using either Spearman or Pearson correlation coefficients as implemented within the respective platforms (GEPIA3, TIMER3.0, and MEXPRESS). Survival analyses were conducted using Kaplan-Meier estimation with log-rank testing through the TCGA and CGGA survival analysis portals.

Unless otherwise specified by the platform, a p-value < 0.05 was considered statistically significant. Data verification, formatting, and figure preparation were performed using R (version 4.5.2) with commonly used packages including ggplot2, dplyr, and tidyr.

All web-based analyses were performed using publicly accessible platforms, including GEPIA3, UALCAN, TIMER3.0, STRING (version 12), and MEXPRESS. These analyses were conducted between October and November 2025 using the default analytical settings provided by each platform unless otherwise specified. For immune infiltration analysis, correlations were examined across multiple immune deconvolution algorithms implemented within TIMER3.0 (including TIMER, CIBERSORT, quanTIseq, EPIC, xCell, and MCP-counter), and consistent trends observed across algorithms were reported. Because these analyses rely on web-based resources that may be periodically updated, exact replication may depend on the database versions available at the time of access.

## Results

TRAIL decoy receptors demonstrate progressive upregulation across glioma grades

**Figure 1:**
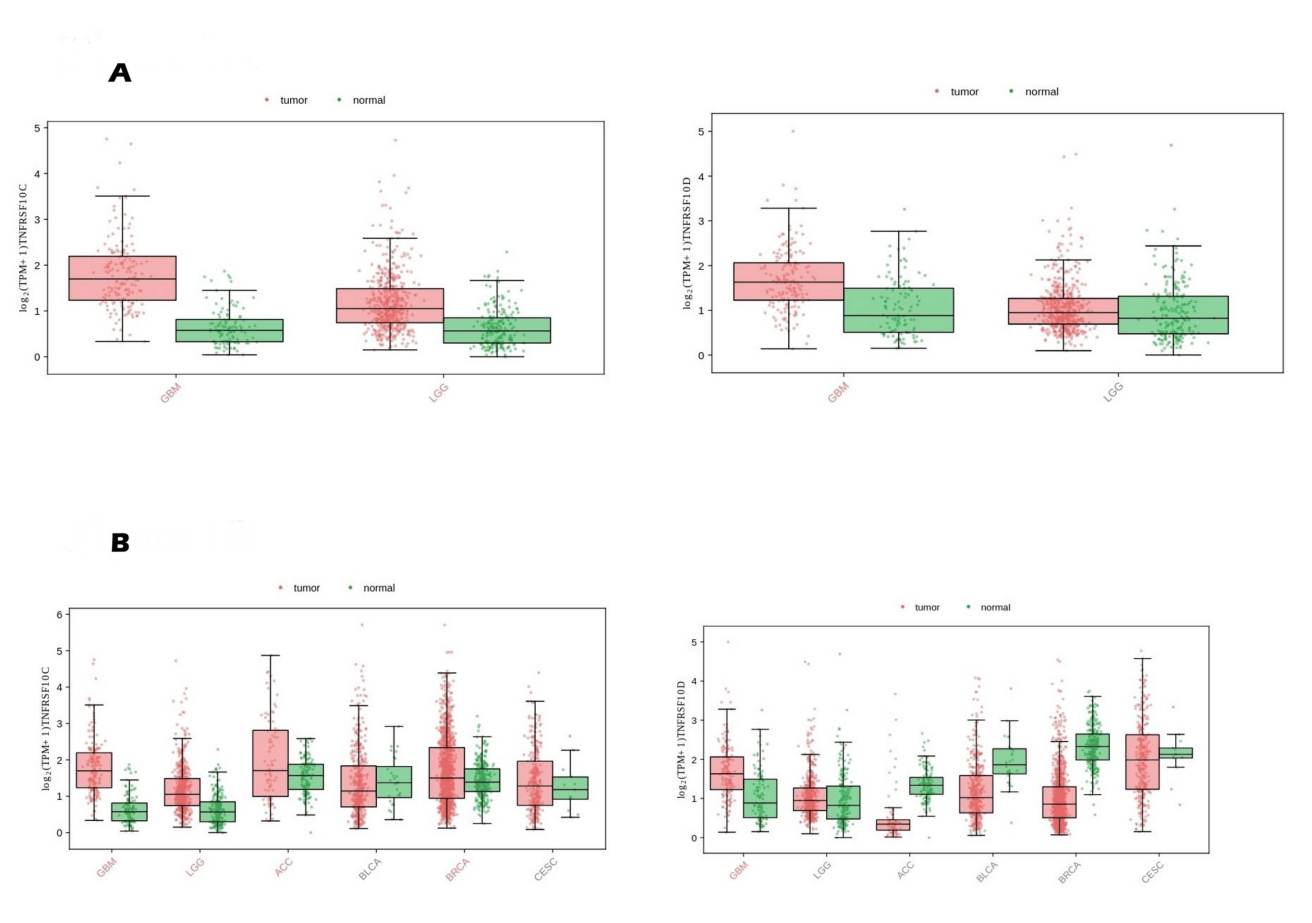
Baseline transcriptional expression patterns of TRAIL decoy receptors in gliomas and across human cancers TRAIL: tumor necrosis factor-related apoptosis-inducing ligand; GBM: glioblastoma; LGG: lower-grade glioma; TCGA: The Cancer Genome Atlas; GTEx: genotype-tissue expression; TNFRSF10C and TNFRSF10D: tumor necrosis factor receptor superfamily member 10C and 10D (A) Expression of TNFRSF10C and TNFRSF10D in GBM and LGG compared with normal brain tissues using GEPIA3 (TCGA + GTEx datasets). Each gene is displayed in a separate panel with its own expression scale, reflecting gene-specific expression distributions. (B) Pan-cancer comparison of TNFRSF10C and TNFRSF10D expression across TCGA tumor samples and GTEx normal tissues

Expression patterns of TNFRSF10C and TNFRSF10D were evaluated across normal brain tissue and glioma subtypes using GEPIA3. Each gene was analyzed and visualized independently, resulting in gene-specific expression distributions across normal brain, lower-grade glioma (LGG), and glioblastoma (GBM). Both genes were significantly dysregulated in GBM relative to normal tissue. Extending the analysis across the glioma continuum (TCGA-LGG, TCGA-GBM, GTEx normal), TNFRSF10C increased from approximately 0.58 in normal brain to 1.05 in LGG and 1.70 in GBM (LGG vs normal p = 1.47 × 10⁻²⁸; GBM vs normal p = 3.10 × 10⁻³⁵). TNFRSF10D showed minimal change from normal to LGG (0.82 → 0.95; p = 0.07) but increased in GBM (1.63; p = 2 × 10⁻¹²). Expression levels of TNFRSF10C and TNFRSF10D were positively correlated across samples, suggesting coordinated expression patterns (Figure 1).

Increased expression in IDH-wildtype gliomas

**Figure 2:**
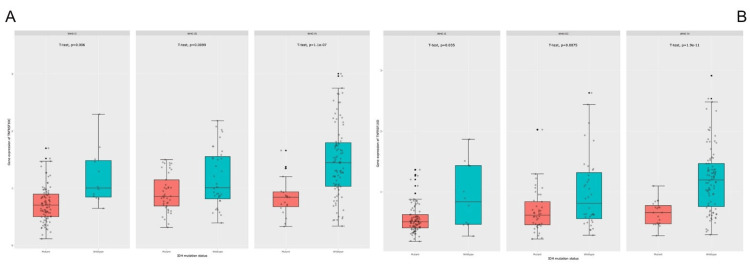
Differential expression of TNFRSF10C and TNFRSF10D according to IDH mutation status in gliomas (CGGA mRNAseq_325 cohort) TNFRSF10C and TNFRSF10D: tumor necrosis factor receptor superfamily member 10C and 10D; CGGA: Chinese Glioma Genome Atlas; IDH: isocitrate dehydrogenase

Using the CGGA mRNAseq_325 dataset, expression levels were evaluated across IDH-defined molecular subgroups. Both genes demonstrated higher expression in IDH-wildtype tumors across WHO grades. For TNFRSF10C, higher expression in wildtype tumors was observed in grade II (p = 0.006), grade III (p = 0.0099), and grade IV GBM (p = 1.1 × 10⁻⁷). TNFRSF10D showed similar patterns in grade II (p = 0.035), grade III (p = 0.0075), and grade IV (p = 1.9 × 10⁻¹¹) (Figure 2).

Promoter methylation patterns and expression associations

**Figure 3:**
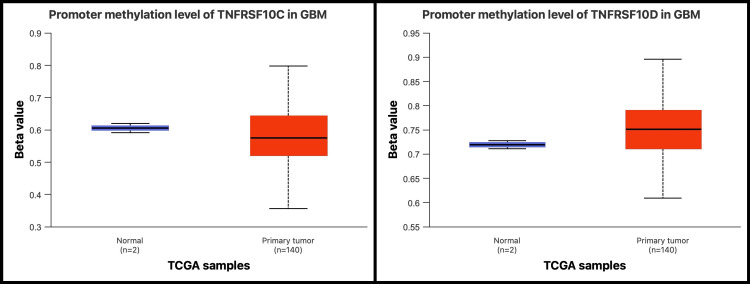
Promoter methylation patterns of TNFRSF10C and TNFRSF10D in GBM GBM: glioblastoma; TCGA: The Cancer Genome Atlas; TNFRSF10C and TNFRSF10D: tumor necrosis factor receptor superfamily member 10C and 10D Boxplots comparing promoter methylation β-values of TNFRSF10C and TNFRSF10D between TCGA normal brain tissue (n = 2) and primary GBM samples (n = 140)

UALCAN-based analysis showed that promoter methylation levels for TNFRSF10C and TNFRSF10D were higher in GBM samples compared with normal brain tissue (Figure 3). This analysis reflects a group-level comparison between tumor and normal tissues.

**Figure 4:**
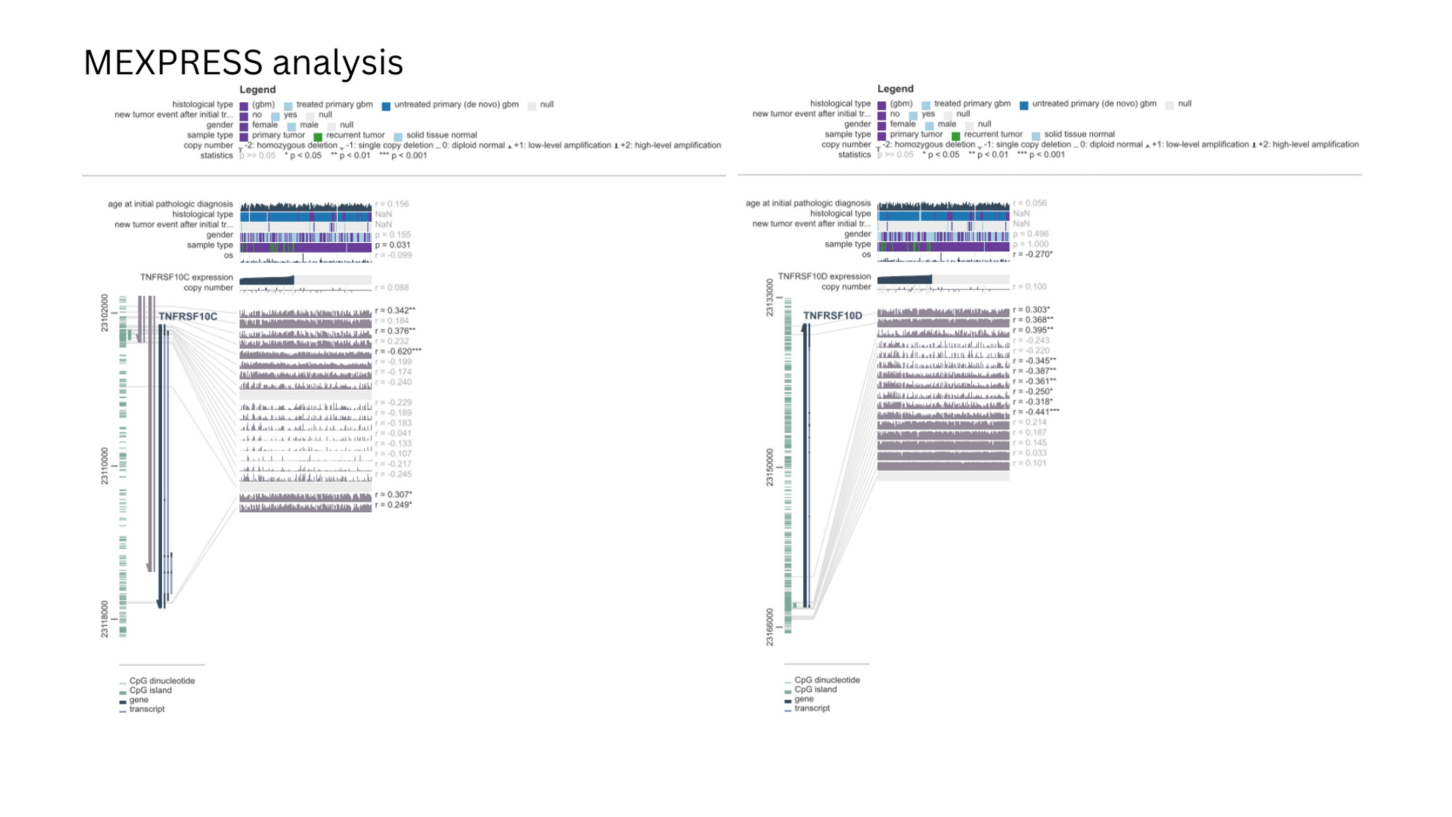
MEXPRESS analysis GBM: glioblastoma; TCGA: The Cancer Genome Atlas; TNFRSF10C and TNFRSF10D: tumor necrosis factor receptor superfamily member 10C and 10D MEXPRESS visualization of TNFRSF10C (left) and TNFRSF10D (right) across the TCGA-GBM cohort. CpG probes are mapped along genomic coordinates with corresponding β-values, clinical covariates, and correlation coefficients with gene expression. Multiple promoter-proximal CpG sites demonstrate inverse correlations (r < 0) with transcript levels

To further examine the relationship between promoter methylation and gene expression, CpG-level correlation analysis was performed using MEXPRESS within the TCGA-GBM cohort (Figure 4). Several promoter-associated CpG sites demonstrated inverse correlations with gene expression. For TNFRSF10C, promoter probes showed negative correlations (cg20560881 r ≈ −0.47, p ≈ 1 × 10⁻⁹; cg24478635 r ≈ −0.43, p ≈ 3 × 10⁻⁸; cg08180290 r ≈ −0.42, p ≈ 7×10⁻⁸). Similar inverse correlations were observed for TNFRSF10D (cg14418122 r ≈ −0.49, p ≈ 3 × 10⁻¹⁰; cg04362553 r ≈ −0.47, p ≈ 9 × 10⁻¹⁰; cg12785352 r ≈ −0.46, p ≈ 1 × 10⁻⁹).

These analyses represent different analytical contexts. The UALCAN results describe promoter methylation differences between GBM and normal brain tissues, whereas the MEXPRESS analysis evaluates methylation-expression correlations across individual GBM samples. Together, these findings indicate that promoter CpG methylation patterns are associated with variability in gene expression within GBM tumors.

Genomic alterations show a limited mutation burden

Mutation analysis indicated that TNFRSF10C and TNFRSF10D are infrequently mutated in glioma cohorts. Copy-number variation analysis identified amplification and shallow deletions at modest frequencies. CNV-associated expression changes were observed, but did not demonstrate strong survival associations. As described in the Methods section, these observations were derived from exploratory inspection of publicly available TCGA visualization resources. They were included as descriptive genomic context rather than as primary analytical results.

Network analysis identifies association with inflammatory signaling components

STRING-based protein-protein interaction (PPI) analysis demonstrated that TNFRSF10C and TNFRSF10D cluster with multiple TRAIL/TNFRSF family members, apoptosis-related proteins, and inflammatory mediators. The interaction network includes proteins such as CASP8, FADD, CFLAR, TNF, and DR4/DR5, indicating that these receptors participate in a broader TRAIL/TNF-associated signaling context rather than representing isolated components.

Immune deconvolution reveals associations with myeloid cell populations

**Figure 5:**
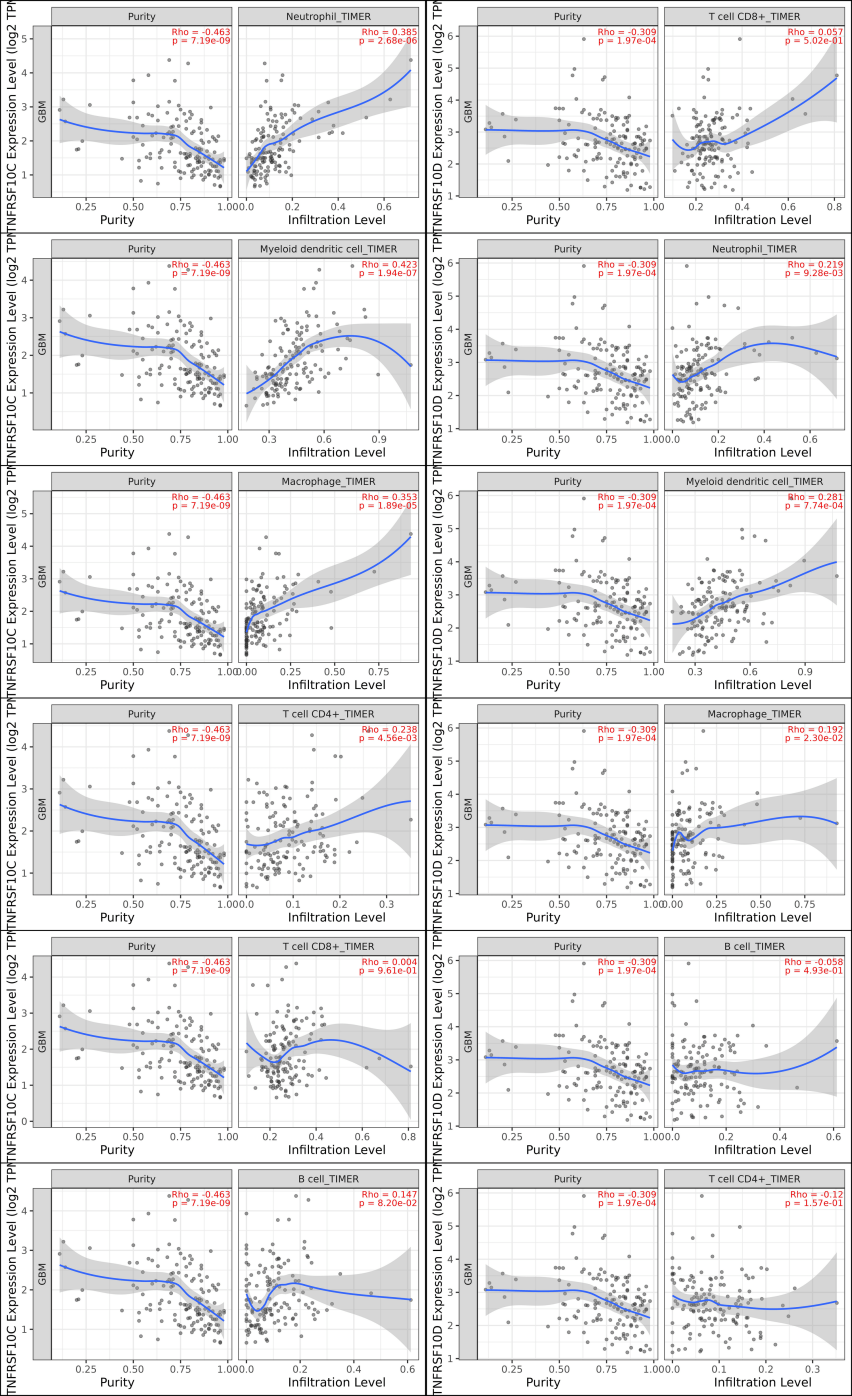
Correlation between TNFRSF10C (DcR1) and TNFRSF10D (DcR2) expression and immune cell infiltration in GBM GBM: glioblastoma; TNFRSF10C and TNFRSF10D: tumor necrosis factor receptor superfamily member 10C and 10D (A) Spearman correlations between TNFRSF10C expression and tumor purity, neutrophils, myeloid dendritic cells, macrophages, CD4⁺ T cells, CD8⁺ T cells, and B cells in GBM. (B) Spearman correlations between TNFRSF10D expression and the same immune cell populations. Shaded areas represent 95% confidence intervals. Correlation coefficients (ρ) and corresponding p-values were calculated using TIMER 3.0 with partial Spearman analysis adjusted for tumor purity

Immune cell abundance was assessed using TIMER3.0 (Figure 5). TNFRSF10C expression showed positive correlations with macrophages (TIMER ρ ≈ 0.35; EPIC ρ ≈ 0.26), neutrophils (CIBERSORT ρ ≈ 0.36; TIMER ρ ≈ 0.38), and dendritic cells (TIMER ρ ≈ 0.42; MCP-counter ρ ≈ 0.30). Correlations with CD8⁺ T cells, CD4⁺ T cells, and B cells were weak or negative. TNFRSF10D demonstrated similar correlation patterns, with the strongest associations observed for dendritic cells (TIMER ρ ≈ 0.28; CIBERSORT ρ ≈ 0.14) and moderate associations with neutrophils and macrophages.

Clinical outcome analysis

**Figure 6:**
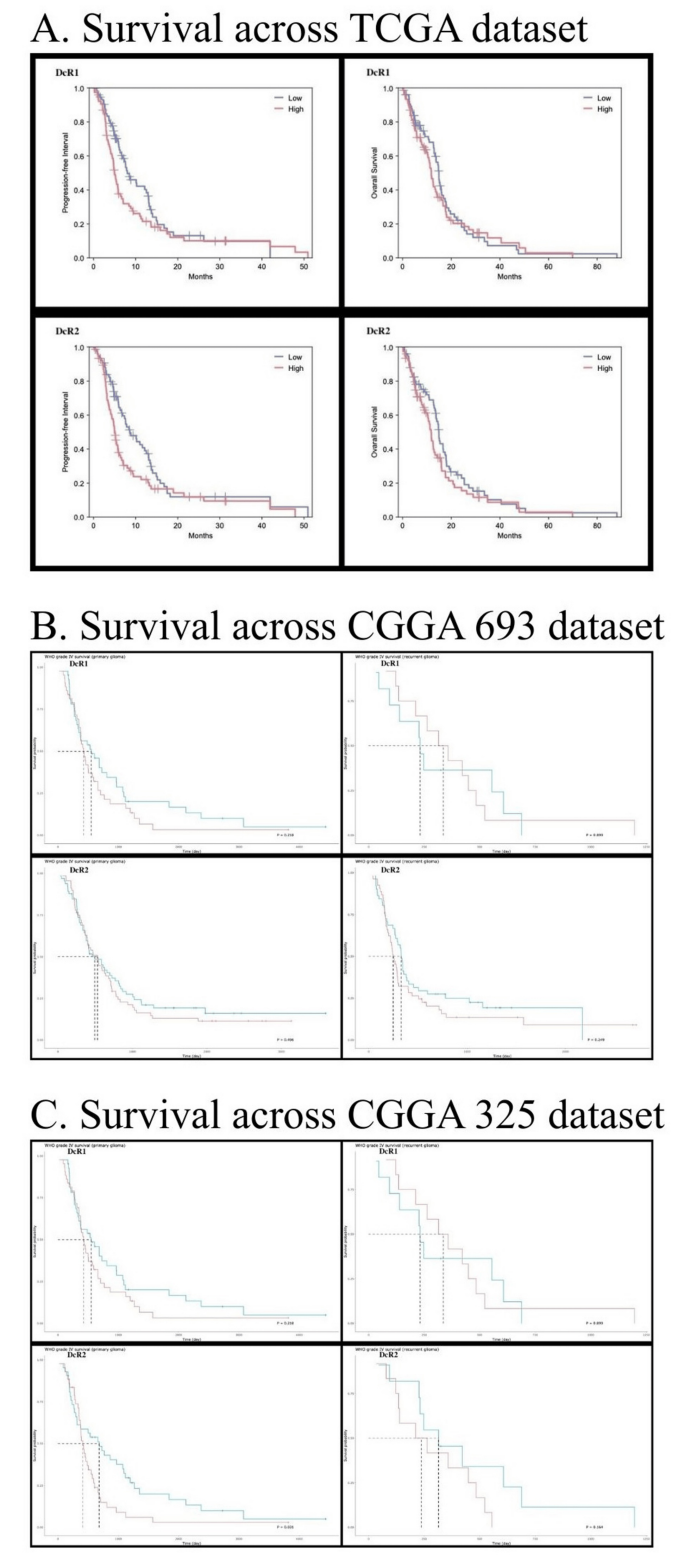
Survival analysis of TNFRSF10C (DcR1) and TNFRSF10D (DcR2) across independent glioma cohorts. TNFRSF10C and TNFRSF10D: tumor necrosis factor receptor superfamily member 10C and 10D; CGGA: Chinese Glioma Genome Atlas; GBM: glioblastoma; TCGA: The Cancer Genome Atlas (A) TCGA-GBM cohort: Overall survival (OS) and progression-free interval (PFI) stratified by high versus low expression of TNFRSF10C and TNFRSF10D.  (B) CGGA693 cohort: Kaplan-Meier survival curves for TNFRSF10C and TNFRSF10D expression groups in primary and recurrent GBM samples. (C) CGGA325 cohort: Survival analysis of TNFRSF10C and TNFRSF10D in an independent validation dataset. Group stratification was based on median expression levels. Statistical significance was assessed using log-rank testing

Kaplan-Meier survival analyses were performed across TCGA and CGGA cohorts to evaluate the prognostic relevance of TNFRSF10C and TNFRSF10D expression (Figure 6). In the TCGA-GBM cohort, TNFRSF10D expression was not significantly associated with overall survival (OS) (HR = 1.30; 95% CI: 0.91-1.87; log-rank p = 0.152). However, higher TNFRSF10D expression was associated with shorter progression-free interval (PFI) (HR = 1.53; 95% CI: 1.06-2.20; p = 0.0218). TNFRSF10C expression did not demonstrate a significant association with OS (HR = 1.14; 95% CI: 0.80-1.64; p = 0.458), while a nonsignificant trend toward shorter PFI was observed (HR = 1.42; 95% CI: 0.98-2.04; p = 0.0604).

In the CGGA mRNAseq_325 cohort, TNFRSF10D expression demonstrated a statistically significant association with survival in primary glioma samples (log-rank p = 0.031), whereas no significant association was observed in recurrent tumors (p = 0.16). TNFRSF10C expression did not show statistically significant survival associations in either primary (p = 0.22) or recurrent tumors (p = 0.90).

Similarly, in the CGGA mRNAseq_693 cohort, TNFRSF10D expression was not significantly associated with survival in primary (p = 0.26) or recurrent tumors (p = 0.19). TNFRSF10C expression also showed no significant survival association in primary (p = 0.50) or recurrent samples (p = 0.25).

Overall, survival analyses across multiple independent cohorts suggest that while TNFRSF10D expression may show modest associations with disease progression in certain datasets, neither receptor demonstrates consistent prognostic effects across glioma cohorts.

Summary of integrated findings

**Figure 7:**
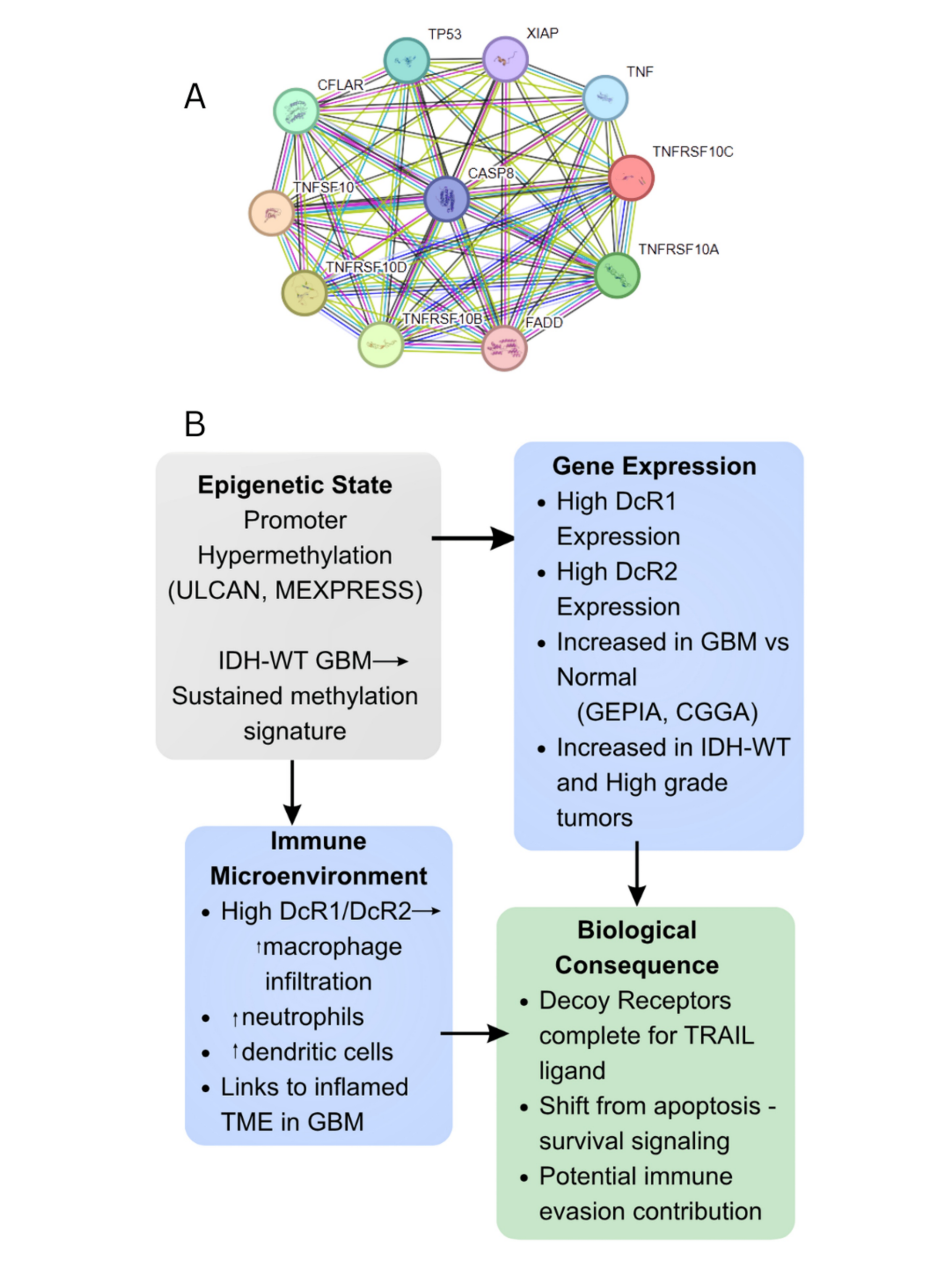
Integrated overview of TNFRSF10C (DcR1) and TNFRSF10D (DcR2) expression patterns and associated pathways in GBM TRAIL: tumor necrosis factor-related apoptosis-inducing ligand; GBM: glioblastoma; TNFRSF10C and TNFRSF10D: tumor necrosis factor receptor superfamily member 10C and 10D; IDH: isocitrate dehydrogenase (A) Protein-protein interaction (PPI) network generated using STRING showing interactions between proteins associated with TNFRSF10C and TNFRSF10D. In the STRING network, nodes represent proteins and edges represent functional or physical interactions derived from curated databases, experimental data, and computational predictions. The network highlights connections with apoptosis-related and cytokine-associated proteins, including CASP8, FADD, CFLAR, TNF, and DR4/DR5. (B) Schematic representation summarizing observed multi-layer associations. Promoter methylation patterns (UALCAN, MEXPRESS) and transcriptomic analyses indicate differential expression of TNFRSF10C and TNFRSF10D in GBM, particularly in IDH-wildtype and higher-grade tumors. Immune deconvolution analyses (TIMER) demonstrate associations between receptor expression and myeloid cell populations, including macrophages, neutrophils, and dendritic cells. The diagram illustrates the established biological role of DcR1/DcR2 as decoy receptors within TRAIL signaling This image was created using Canva (Canva Pty Ltd.; Sydney, Australia)

Across transcriptomic, epigenetic, genomic, immune, and clinical analyses, TNFRSF10C and TNFRSF10D demonstrated progressive upregulation with tumor grade, higher expression in IDH-wildtype gliomas, inverse correlation with promoter methylation, and positive associations with myeloid immune cell populations. These findings collectively describe consistent expression and immune-associated patterns in glioblastoma (Figure 7).

## Discussion

GBM remains a highly treatment-resistant malignancy characterized by genetic alterations, immune dysregulation, and epigenetic changes. In this context, the TRAIL decoy receptors TNFRSF10C and TNFRSF10D have historically been viewed primarily as inhibitors of apoptosis. Our multi-omic analysis suggests that their expression patterns in glioma are associated with tumor grade, IDH status, promoter methylation, and immune microenvironment features.

Both receptors demonstrated progressive upregulation from normal brain tissue to LGG and GBM, consistent with increasing tumor grade. Their higher expression in IDH-wildtype tumors further aligns with the more aggressive molecular subtype of glioma. Promoter methylation analyses revealed inverse correlations between CpG methylation and transcript levels, supporting an association between promoter methylation patterns and receptor expression [19,20]. The coexistence of promoter methylation signals and elevated transcript abundance at the cohort level highlights the complexity of transcriptional regulation in GBM.

Immune deconvolution analyses identified consistent associations between TNFRSF10C and TNFRSF10D expression and myeloid cell populations, including macrophages, neutrophils, and dendritic cells, whereas correlations with lymphoid subsets were limited. This pattern parallels the established myeloid-predominant microenvironment of GBM [21,22]. Although these analyses do not establish causality, they suggest that decoy receptor expression is linked to inflammatory and innate immune-associated contexts within the tumor microenvironment. Protein-protein interaction network analysis further places these receptors within an interaction context involving TRAIL/TNF-associated signaling components, consistent with their known biological roles.

Survival analyses did not demonstrate strong or consistent associations with overall survival across independent cohorts. However, modest trends toward shorter progression-free intervals were observed in higher-expression groups. These findings indicate that TNFRSF10C and TNFRSF10D may be better interpreted as contextual biomarkers reflecting tumor state rather than as independent prognostic drivers [23-25].

Several limitations should be acknowledged. Bulk RNA sequencing and computational immune deconvolution cannot resolve cell-type-specific expression or spatial relationships within the tumor microenvironment. Additionally, correlation-based analyses cannot determine whether decoy receptor expression actively influences immune interactions or reflects broader inflammatory signaling. Future studies incorporating single-cell and spatial transcriptomic approaches will be required to clarify these relationships.

In summary, this integrative analysis identifies consistent associations between TNFRSF10C and TNFRSF10D expression, epigenetic features, tumor grade, IDH status, and myeloid immune infiltration in GBM. These findings support further investigation into the potential biological relevance of TRAIL decoy receptors within the GBM microenvironment.

## Conclusions

TNFRSF10C and TNFRSF10D demonstrate consistent associations with tumor grade, IDH status, promoter methylation patterns, and myeloid-predominant immune infiltration in GBM. While these findings do not establish causality, they highlight reproducible expression and immune-related trends across independent datasets. Together, the results support further investigation into the potential biological and microenvironmental relevance of TRAIL decoy receptors in GBM.
